# Supplementary material for: Modeling glioblastoma heterogeneity as a dynamic network of cell states
Source: Mol Syst Biol. 2021 Sep 16;17(9):e10105. doi: 10.15252/msb.202010105 (PMC8444284; doi:10.15252/msb.202010105)
Supplement: Supplementary file 6 — Source Data for Figure 5 [file MSB-17-e10105-s004.zip › Figure5A_sourcedata/GSEA_3017/hallmarks_stateC.GseaPreranked.1621934520804/HALLMARK_IL2_STAT5_SIGNALING.html]

Details for gene set HALLMARK\_IL2\_STAT5\_SIGNALING[GSEA]

|  || Dataset | state13017 |
| Phenotype | NoPhenotypeAvailable |
| Upregulated in class | na\_neg |
| GeneSet | HALLMARK\_IL2\_STAT5\_SIGNALING |
| Enrichment Score (ES) | -0.52190447 |
| Normalized Enrichment Score (NES) | -2.3776855 |
| Nominal p-value | 0.0 |
| FDR q-value | 0.0021846765 |
| FWER p-Value | 0.005 |
Table: GSEA Results Summary

  

Fig 1: Enrichment plot: HALLMARK\_IL2\_STAT5\_SIGNALING      
 Profile of the Running ES Score & Positions of GeneSet Members on the Rank Ordered List

  

| PROBE | GENE SYMBOL | GENE\_TITLE | RANK IN GENE LIST | RANK METRIC SCORE | RUNNING ES | CORE ENRICHMENT || 1 | CCND2 |  |  | 149 | 0.288 | -0.2310 | No |
| 2 | ARL4A |  |  | 224 | -0.259 | -0.3341 | No |
| 3 | PHLDA1 |  |  | 295 | -0.274 | -0.4286 | No |
| 4 | BATF3 |  |  | 318 | -0.284 | -0.4384 | No |
| 5 | CSF1 |  |  | 320 | -0.285 | -0.4115 | No |
| 6 | AHR |  |  | 324 | -0.286 | -0.3879 | No |
| 7 | RHOB |  |  | 391 | -0.316 | -0.4713 | No |
| 8 | MYC |  |  | 421 | -0.336 | -0.4881 | Yes |
| 9 | SLC2A3 |  |  | 422 | -0.336 | -0.4542 | Yes |
| 10 | KLF6 |  |  | 429 | -0.339 | -0.4305 | Yes |
| 11 | WLS |  |  | 438 | -0.345 | -0.4097 | Yes |
| 12 | SERPINB6 |  |  | 463 | -0.367 | -0.4146 | Yes |
| 13 | ALCAM |  |  | 467 | -0.370 | -0.3825 | Yes |
| 14 | NT5E |  |  | 500 | -0.416 | -0.3964 | Yes |
| 15 | CA2 |  |  | 518 | -0.448 | -0.3809 | Yes |
| 16 | AHNAK |  |  | 519 | -0.449 | -0.3357 | Yes |
| 17 | TNFRSF21 |  |  | 526 | -0.461 | -0.2997 | Yes |
| 18 | IFITM3 |  |  | 533 | -0.477 | -0.2621 | Yes |
| 19 | COL6A1 |  |  | 534 | -0.479 | -0.2139 | Yes |
| 20 | ITGA6 |  |  | 535 | -0.479 | -0.1657 | Yes |
| 21 | HOPX |  |  | 546 | -0.513 | -0.1314 | Yes |
| 22 | CD44 |  |  | 575 | -0.659 | -0.1139 | Yes |
| 23 | SPP1 |  |  | 577 | -0.674 | -0.0477 | Yes |
| 24 | EMP1 |  |  | 589 | -0.785 | 0.0122 | Yes |
Table: GSEA details [plain text format]

  

Fig 2: HALLMARK\_IL2\_STAT5\_SIGNALING: Random ES distribution      
 Gene set null distribution of ES for **HALLMARK\_IL2\_STAT5\_SIGNALING**
